# Prognostic Value of N-Terminal Pro-B-Type Natriuretic Peptide and Glomerular Filtration Rate in Patients With Acute Heart Failure

**DOI:** 10.3389/fcvm.2020.00123

**Published:** 2020-07-21

**Authors:** Kai Wang, Gehui Ni, Qianyun Wu, Yanli Zhou, Wenming Yao, Haifeng Zhang, Xinli Li

**Affiliations:** Department of Cardiology, The First Affiliated Hospital of Nanjing Medical University, Nanjing, China

**Keywords:** acute heart failure (AHF), N-terminal pro-B-type natriuretic peptide (NT-proBNP), glomerular filtration rate (GFR), outcomes, prognosis

## Abstract

**Aims:** To investigate the relationship between N-terminal pro-B-type natriuretic peptide (NT-proBNP), Glomerular Filtration Rate (GFR), and outcomes in patients hospitalized with acute heart failure (AHF).

**Methods:** The trial was registered at http://www.chictr.org/cn/. (ChiCTR – ONC - 12001944). A total of 493 patients hospitalized for AHF in cardiology department of the First Affiliated Hospital of Nanjing Medical University from March 2012 to October 2016 were enrolled into registry. The end event was the occurrence of all-cause death within an 18-month follow-up. The data collected from the participants in admission were used to calculate the GFR by chronic kidney disease epidemiology collaboration equation (CKD-EPI) and performed the according statistical analysis.

**Results:** There were 74 participants (13.8%) dropped out and 91 (21.7%) passed away within the 18-month follow up. Comparison of clinical indicators between survival and death group were analyzed for the long-term prognosis of patients with AHF. In the single factor analysis, both NT-proBNP and GFR were statistically significant (*P* < 0.001). Combined NT-proBNP and GFR in multi-factor COX regression analysis showed significant predictive value (*P* < 0.001). In receiver operator characteristics (ROC) analyses, the area under the curves (AUC) for NT-proBNP was 0.648 [95%CI: 0.598–0.695, *P* < 0.001] and for GFR was 0.677 [95%CI: 0.627–0.723, *P* < 0.001]. According to the Youden index, the best prediction point of NT-proBNP was 2,137 pg/ml and GFR was 61.7 ml/(min·1.73 m^2^). After using the Binary Logistic Regression to combine the two indicators, the AUC was 0.711, which was significantly compared to the AUC of either single factor. The sensitivity of the combined indicators were 0.535, the specificity were 0.853. According to the cut-off point, these two indexes were separated into four groups for further analysis by Kaplan-Meier survival curve comparison (log-rank test), which showed that patients in the group with higher NT-proBNP and lower GFR had the worst prognosis.

**Conclusions:** In patients with NT-proBNP > 2,137 pg/ml and GFR < 61.7 ml/(min·1.73 m^2^), the risk of death was significantly higher. The combination of GFR and NT-proBNP improved the predictive value for the long-term prognosis of AHF patients.

## Introduction

Heart failure (HF) is one of the common diseases of the cardiovascular system. The current annual incidence of HF in the United States and Europe are more than one million (1, 2). Acute heart failure (AHF) refers to the insufficient tissue perfusion and acute systemic or pulmonary stasis syndrome caused by a sharp decrease in cardiac output. It is clinically dangerous and progresses rapidly, which is likely to cause systemic hemodynamic disorder and multi-organ failure (3). All-cause death of AHF patients were recorded in 10% at 30-day follow-up and 50.1% at 1-year follow-up (4). In recent years, new ideas have been proposed for the treatment of HF, such as exercise, microRNAs, etc. (5, 6). However, the prognosis of hospitalized patients with HF is relatively limited. With the deepening of the research on the pathogenesis of AHF, the application value of biomarkers in the early diagnosis, risk stratification, and prognosis assessment of AHF has become the focus of research. N-terminal pro-B-type natriuretic peptide (NT-proBNP) is widely used as a clinical AHF prediction factor. Due to the large number of interfering factors, the prognosis of patients with complex HF evaluated by NT-proBNP alone has limits, which results in an urgent need for a more effective method in clinical practice. In this study we evaluated renal function indicators in patients with AHF. To provide more information about AHF prognosis to medical worker, the new CKD-EPI formula was used to calculate the glomerular filtration rate (GFR) and to evaluate the long-term survival rate of AHF patients combined with NT-proBNP.

In recent years, NT-proBNP measurement has been widely recognized as an auxiliary diagnostic condition for AHF (7). Currently in the clinical practice guidelines, it is included in the level I recommendation, evidence level: A (8–10). However, some studies have shown that the interference factors of NT-proBNP level are various, including race, sex (higher in females), obesity, anemia, common kidney disease, atrial fibrillation, chemotherapy drugs, and enkephalinase inhibitor drug (10). The interaction between heart diseases and kidney diseases is a hot topic in recent years. To elucidate the interaction between heart function and kidney function, Ledoux first proposed the concept of “cardiorenal syndrome” (CRS), kidney injury induced by HF, in 1951 (11). In 2005, Bongartz et al. redefined CRS as the dysfunction of one organ in the heart and kidneys leading to acute or chronic insufficiency of the other (12). When both heart insufficiency and renal insufficiency occur at the same time, the fatality rate will increase significantly. In this paper, we mainly discuss with type I CRS (AHF with acute kidney injury), which is common in patients hospitalized for acute decompensated HF with an incidence up to 25% (13, 14). Especially in the acute stage, the interaction between cardiac and renal functions causes more serious clinical events and deteriorates the prognosis of patients. For those patients, the potential for kidney damage should be identified timely for intervention as soon as possible in the treatment of AHF.

As a recognized independent prognostic factor, NT-proBNP has a higher sensitivity, but its specificity is affected by many non-cardiac factors, which often brings certain limitations to clinical diagnosis and evaluation. At present, for most countries in the world, medical workers analyze the condition and prognosticate AHF by using the way that combines cardiac troponin I/T(cTnI/T), hemoglobin, cystatin, and others in clinical practice (15–18). In this study, renal related markers were analyzed to explore the prognostic value of NT-proBNP combined with GFR for long-term survival in AHF patients. We adopted a prospective method by enrolling 493 AHF patients, collecting their clinical data, and ending the 18-month follow-up on time. Finally, SPSS and MedCalc statistical software were used to analyze the clinical data of 419 AHF patients who completed 18-month follow-up. It was concluded that the combination of GFR and NT-proBNP can effectively predict the long-term survival of AHF patients and improve the predictive value of NT-proBNP on mortality.

## Methods

### Subjects

A total of 493 patients hospitalized for AHF (including the initial onset of AHF and acute decompensated HF) in Cardiology Department of The First Affiliated Hospital of Nanjing Medical University from March 2012 to October 2016 were enrolled into registry. The diagnosis of AHF was referred to the guidelines for AHF in China. Inclusion criteria: (1) Age ≥ 18 years old, gender is not limited; (2) The onset of AHF or CHF usually includes symptoms and signs of pulmonary congestion, systemic congestion or cardiac output reduction, the NT-proBNP > 300 pg/ml, and echocardiography indicates abnormalities in cardiac structure or function; (3) Subjects should participate in the study voluntarily and sign the informed consent. Exclusion criteria: (1) Patients with malignant tumors; (2) Patients with cognitive impairment and dementia; (3) Patients with severe hepatorenal insufficiency, and primary chronic nephropathy; (4) Patients with other serious uncontrollable systemic diseases; (5) Patients who were unwilling to sign the informed consent forms or were unable to complete all follow-up. This study has been approved by the hospital ethics committee, follows the principles of clinical practice and the Helsinki declaration, and requires each enrolled patient to sign an informed consent form.

### Data Collection

Clinical data of enrolled patients were collected and basic database was established. After enrollment, the patient's hospitalization history was checked, and their age, gender, contact information, height, weight, and other basic information were recorded. Besides, patient's admission diagnosis, etiology of AHF and history of concomitant diseases such as hypertension, diabetes, myocardial infarction, atrial fibrillation, renal insufficiency, and thyroid dysfunction were recorded. At the same time, the routine examination results of the patients on admission were collected, including blood routine, blood biochemistry, NT-proBNP, myocardial markers, coagulation function, routine 12-lead electrocardiogram, echocardiography, dynamic electrocardiogram, chest film, etc., and the diagnosis and treatment in the hospital and drug regimen were recorded.

### Specimen Collection and Detection

For all patients signed the informed consent, the blood was collected through the cubital vein and injected into the anticoagulant tubes and coagulant tubes with patients' limosis condition on the morning of the second day after admission. All serological tests were completed by the laboratory division of our hospital using AU 5800 automatic biochemical analyzer (Beckman Coulter, USA) and an automatic analyzer for NT-proBNP (Roche Elecsys®proBNP immunoassay, Switzerland). The unit of NT-proBNP is pg/mL and the unit of GFR is ml/(min·1.73 m^2^).

### Ckd-Episcr Formula Was Used for GFR Calculation (19)

1. Women: ①serum creatinine ≤ 0.7 mg/dl, GFR = 144 × (serum creatinine (mg/ dl)/0.7)^−0.329^ × (0.993)^age^; the serum creatinine > 0.7 mg/dl, GFR = 144 × (serum creatinine (mg/dl)/0.7)^−1.209^ × (0.993)^age^.
2. Men: ①serum creatinine ≤ 0.9 mg/dl, GFR = 141 × (serum creatinine (mg/dl)/0.9)^−0.411^ × (0.993)^age^; serum creatinine > 0.9 mg/dl, GFR = 141 × (serum creatinine (mg/dl)/0.9)^−1.209^ × (0.993)^age^.

Creatinine units conversion: 1 mg/dl = 88.4 μmol/l.

### Routine 12-Lead Electrocardiogram and Echocardiography

The routine 12-lead electrocardiogram was completed by qualified clinicians with FS-8322 12-channel automatic analysis electrocardiogram machine from Beijing Futian electronic medical instrument Co., LTD.

Echocardiograph was collected by VIVID E9 color doppler ultrasonography (GE, USA). The probe frequency was 3.5 mhz, and the four-chamber heart section of the apex was taken. The left ventricular ejection fraction, left ventricular diastolic diameter, left ventricular systolic diameter, and pulmonary artery systolic pressure were measured.

### Follow-Up

Patients were treated normatively by professional cardiologists during hospitalization according to the 2009 and 2014 Chinese Guidelines on Diagnosis and Treatment of AHF, and provided the optimized treatment plans before hospital discharge. Follow-up was conducted by telephone or outpatient service every 3 months after discharge, which was mainly to evaluate the occurrence of end-point events and to record the cause and time of end-point events. No intervention was performed during the follow-up. The total follow-up time was 18 months and the outcome event was all-cause death.

### Statistical Analysis

All data were processed by SPSS 22.0 and MedCalc 11.4.2.0 statistical software. Kolmogorov-smironov (k-s) was used to test the normality of measurement data. The data of normal distribution were expressed as mean±standard deviation, and independent sample *t*-test was used to analyze the difference between two groups. The data of non-normal distribution were represented as median (M) and range (Q1-Q3), and the differences between groups were compared by non-parametric tests. Enumeration data were expressed as frequency or rate, and *x*^2^ test was used for differences between groups. Multiple Cox stepwise regression analysis (forward) was used to identify independent predictors of 18-months mortality in the study. Variables associated with 18-months mortality in univariate analysis (*P* < 0.05) were selected to be adjusted. The specificity and sensitivity of each indicator in the diagnosis of AHF patients' death were calculated by combining receiver operator characteristics (ROC) curve. Kaplan-meier method was used to draw the survival curve, and log-rank test was used for comparison between groups. *P* < 0.05 was considered statistically significant.

## Results

### Basic Population Data

Among the 493 patients enrolled in this study, 74 patients (13.8%) failed to complete the follow-up due to midway withdraw, and a total of 419 patients completed the follow-up. Ninety-one patients (21.7%) died during the 18-month follow-up period. The mean age of follow-up patients was 60.9 ± 15.7 years, and 277 patients (66.1%) were male. HF is classified according to the New York heart association (NYHA) classification standard. Patients were selected according to the NYHA heart function classification: 67 cases of cardiac function level II, 227 cases of cardiac function level III, and 125 cases of cardiac function level IV. According to the occurrence of all-cause death during the 18 months follow-up, the enrolled patients were divided into the death group and the non-death group for test comparison. Detailed results are shown in Table 1. Statistical analysis of 36 variables showed that compared with survival group, the age, NT-proBNP, D-dimer, serum uric acid, urea nitrogen (BUN), aspartate aminotransferase (AST), and pulmonary artery systolic blood pressure (PASP) of the death group were higher; while GFR, systolic pressure, diastolic blood pressure, serum sodium, albumin, and hemoglobin were comparatively lower (*P* < 0.05).

**Table 1:** Baseline clinical characteristics of patients.

	**All patients (*n* = 419)**	**Survival (*n* = 328)**	**Death (*n* = 91)**	***P*-value**
**Demographic characteristics**
Age, years	60.9 ± 15.7	60.0 ± 15.7	64.1 ± 15.5	0.03
Gender(%) male	277 (66.1)	222 (67.7)	55 (60.4)	0.20
female	142 (33.9)	106 (32.3)	36 (39.5)	
SBP, mmHg	124.8 ± 20.4	127.1 ± 20.4	116.5 ± 18.1	<0.01
DBP, mmHg	77.7 ± 13.6	79.5 ± 13.7	71.2 ± 11.3	<0.01
Heart rate, beat/min	85.7 ± 21.1	86.3 ± 21.1	83.5 ± 21.3	0.25
BMI, kg/m^2^	24.3 ± 4.4	24.4 ± 4.7	24.0 ± 3.4	0.39
**Biochemistry examination**
Hemoglobin, g/dl	13.3 ± 2.1	13.5 ± 2.1	12.6 ± 2.2	<0.01
Albumin, g/dl	3.7 ± 0.5	3.7 ± 0.4	3.6 ± 0.6	0.07
Sodium, mM	139.7 ± 3.9	140.1 ± 3.6	138.2 ± 4.5	<0.001
Potassium, mM	3.96 ± 0.47	3.96 ± 0.45	3.99 ± 0.52	0.474
NT-proBNP, pg/ml	1227.0–5254.0 (2178.0)	1091.5–4643.5 (2035.0)	1662.3–7972.8 (2926.0)	<0.001
BUN, mM	5.8–9.1 (7.1)	5.6–8.5 (6.8)	6.5–11.6 (9.1)	<0.001
GFR, ml/(min·1.73 m^2^)	61.2–96.5 (75.8)	63.9–97.6 (79.3)	42.5–91.1 (62.3)	<0.001
Uric acid, μM	479.5 ± 167.1	463.5 ± 148.8	537.4 ± 212.0	0.003
ALT, U/l	16.8–45.8 (26.0)	16.9–42.9 (26.0)	15.9–51.5 (29.3)	0.632
AST, U/l	22.0–41.9 (28.1)	21.7–39.2 (27.3)	23.3–58.0 (33.8)	0.004
D-dimer, mg/l	0.3–1.5 (0.7)	0.3–1.4 (0.6)	0.5–2.5 (1.1)	<0.001
**Device inspection**				
LVEF, %	30.0–55.3 (38.9)	29.9–53.1 (38.5)	30.4–58.8 (40.5)	0.227
LVEDd, mm	61.9 ± 2.5	61.9 ± 11.9	61.6 ± 14.6	0.85
LVEDs, mm	49.3 ± 14.1	49.5 ± 13.6	48.7 ± 15.9	0.64
QRS duration, ms	129.6 ± 40.2	128.4 ± 40.7	133.9 ± 38.3	0.25
QTc, ms	443.6 ± 93.3	443.7 ± 94.4	443.2 ± 89.9	0.96
PASP, mmHg	44.1 ± 16.6	42.6 ± 15.1	50.0 ± 20.2	0.008
**Oral medication at admission**, ***n*** **(%)**
Loop diuretics	397 (95.2%)	309 (94.8%)	88 (96.7%)	0.449
Aldosterone antagonists	387 (92.8%)	300 (92%)	87 (95.6%)	0.243
Digoxin	174 (41.5%)	136 (41.5%)	38 (41.8%)	0.994
ACEI/ARB	340 (81.5%)	263 (80.7%)	77 (84.6%)	0.392
β-blockers	331 (79.4%)	256 (78.5)	75 (82.4%)	0.417
Aspirin	187 (44.8%)	139 (42.6%)	48 (52.7%)	0.086
**Etiology**, ***n*** **(%)**
CHD	106 (25.3%)	82 (25.0%)	24 (26.4%)	0.790
VHD	109 (26.0%)	86 (26.2%)	23 (25.3%)	0.856
Cardiomyopathy	169 (40.3%)	136 (41.4%)	33 (36.3%)	0.371
**Comorbidity**, ***n*** **(%)**				
Hypertension	203 (48.4%)	164 (50%)	39 (42.9%)	0.228
Diabetes Mellitus	91 (21.7%)	68 (20.7%)	23 (25.3%)	0.352
Atrial fibrillation	152 (36.3%)	118 (36.0%)	34 (37.4%)	0.808
**NYHA class**, ***n*** **(%)**				
NYHA class II	67 (16.0%)	57 (17.4%)	10 (11.0%)	0.162
NYHA class III	227 (54.2%)	181 (55.2%)	46 (50.5%)	
NYHA class IV	125 (29.8%)	90 (27.4%)	35 (38.5%)	

*ACEI, angiotension-converting enzyme inhibitor; ALT, alanine aminotransferase; ARB, angiotension receptor blocker; AST, aspartate transaminase; BMI, body mass index; BUN, blood urea nitrogen; CHD, coronary heart disease; DBP, diastolic blood pressure; LVEF, left ventricular ejection fraction; LVEDd, left ventricular end-diastolic dimension; LVEDs, left ventricular end-systolic dimension; NT-proBNP, N-terminal pro-brain natriuretic peptide; NYHA, New York Heart Association; PASP, pulmonary artery systolic pressure; SBP, systolic blood pressure; VHD, valvular heart disease*.

### Multivariate Cox Stepwise Regression Analysis

Single factor analysis was performed on all variables in the baseline data (Table 1). Results showed that NT-proBNP, GFR, sodium, systolic blood pressure (SBP), diastolic blood pressure (DBP), AST, D-dimer, hemoglobin, BUN, PASP, uric acid, and age were valuable on the prognosis of HF (*P* < 0.05). The data of non-normal distribution were changed by Lg10. Lg(GFR) was combined with Lg(NT-proBNP) in binary Logistic regression equation, and then multivariate COX stepwise regression analysis (forward step, *P* ≤ 0.1 into the equation and *P* > 0.1 out of equation) was performed for the statistically significant variables in baseline data to screen out the independent predictors of all-cause death after 18 months follow-up in AHF patients (*P* < 0.05, Table 2). Results showed that the combination of Lg(GFR) and Lg(NT-proBNP) had significant predictive value for long-term survival prognosis in patients with AHF patients (*P* < 0.001). In addition, SBP, sodium, hemoglobin, and PASP were independent predictors of all-cause mortality at 18 months. The role of these variables in predicting HF had been reported, so it was not further described in this paper (15, 20–22).

**Table 2:** COX stepwise regression analysis (forward step, entry only if *P* ≤ 0.10 and removal only if *P* > 0.10) of mortality on the significant (*P* <0.05) variables in baseline characteristics of patients.

	**β value**	**Std. error**	**HR**	**95%CI**	***P* value**
Lg(GFR)+ Lg(NT-proBNP)	3.194	0.625	24.384	7.163~83.005	<0.001
SBP	−0.020	0.007	0.981	0.968~0.993	0.003
Sodium	−0.056	0.028	0.946	0.895~0.999	0.047
PASP	0.021	0.007	1.022	1.007~1.036	0.003
Hemoglobin	−0.014	0.005	0.981	0.968~0.993	0.003
Lg(D-dimer)	0.498	0.256	1.645	0.996~2.716	0.052

*The data of non-normal distribution were changed by Lg10*.

### ROC Curve Analysis

**Figure 1:**
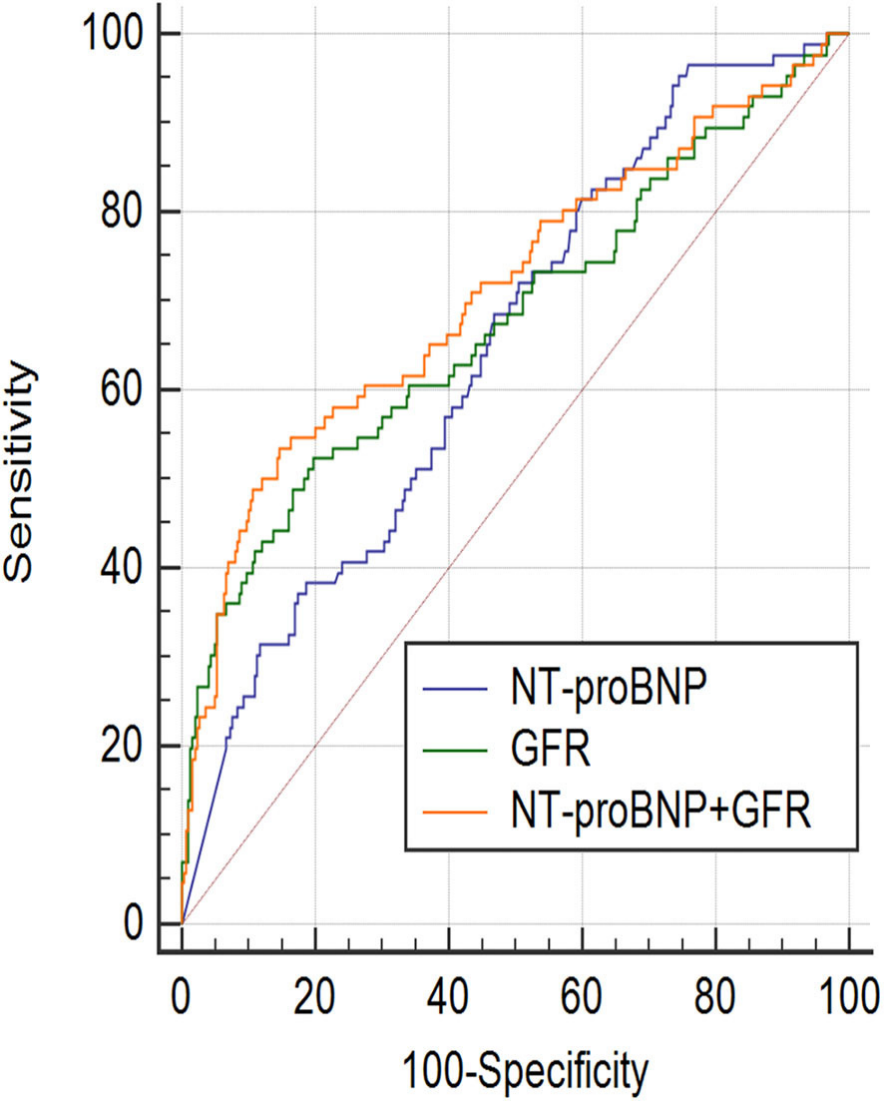
ROC curve (Receiver operator characteristic curves) showed, NT-proBNP and GFR had certain prognosis value of acute heart failure in the 18-month. NT-proBNP combined with GFR had a higher prognostic value. AUC_GFR_ = 0.677, 95%CI: 0.627–0.723, *P* < 0.001; Sensitivity = 49.5%, Specificity = 81.0%, Cut off point: 61.7; AUC_NT−proBNP_ = 0.648, 95%CI: 0.598–0.695, *P* < 0.001; Sensitivity = 68.6%, Specificity = 53.2%, Cut off point: 2,137; AUC_NT−proBNP+GFR_ = 0.711, 95%CI: 0.663–0.756, *P* < 0.001, Sensitivity = 53.5%, Specificity = 85.3%; AUC_GFR_~AUC_NT−proBNP+GFR_, *P* = 0.0117; AUC_NT−proBNP_~AUC_NT−proBNP+GFR_, *P* = 0.0384; AUC_NT−proBNP_~AUC_GFR_, *P* = 0.471.

ROC curve showed that NT-proBNP and GFR had certain value in predicting the prognosis of AHF (AUC_GFR_ = 0.677, 95%CI: 0.627–0.723, *P* < 0.001; AUC_NT−proBNP_ = 0.648, 95%CI: 0.598–0.695, *P* < 0.001). The ROC curves of NT-proBNP and GFR were compared in pairs, and the results showed no statistical difference (*P* > 0.05), as shown in Figure 1. However, the combination of two factors could further improve the AUC (AUC_NT−proBNP+GFR_ = 0.711, 95% CI: 0.663–0.756, *P* < 0.001, sensitivity was 53.5%, specificity was 85.3%), which was significantly different from that of either single factor (Figure 1). According to the ROC curve coordinate and the cutting value of the Yueden index, the optimal prediction value of GFR was 61.7 ml/(min•1.73 m^2^) (sensitivity 49.5%, specificity 81.0%), the optimal prediction value of NT-proBNP was 2,137 pg/ml (sensitivity 68.6%, specificity 53.2%).

### Chi-Square Test and Kaplan-Meier Curve Analysis

**Figure 2:**
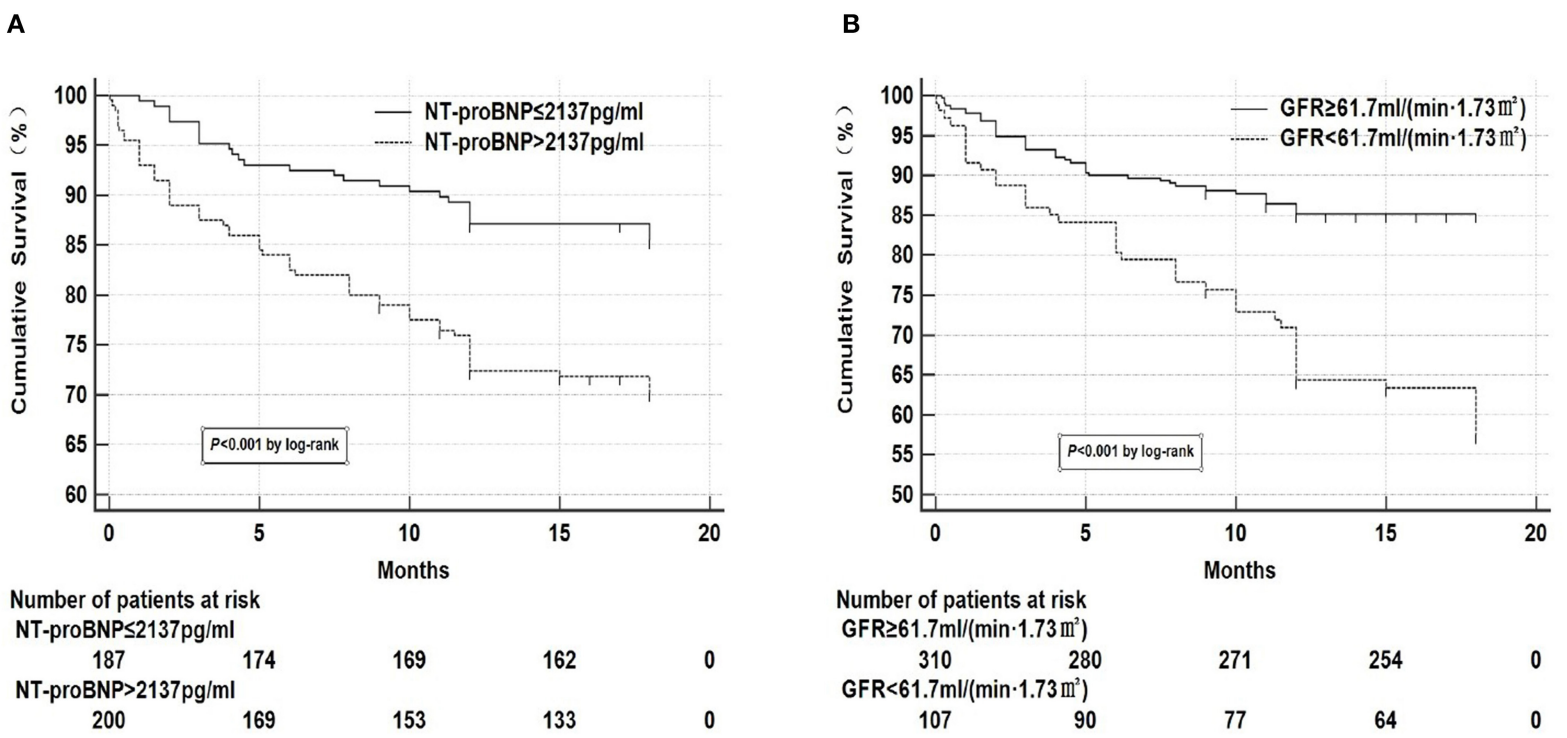
Kaplan-meier curve analysis of NT-proBNP and GFR. **(A)** Kaplan-meier curve analysis showed that mortality significantly increased when NT-proBNP > 2,137 pg/ml. There were 32 missing data for NT-proBNP, 27 in the survival group, and 5 in the death group. **(B)** Kaplan-meier curve analysis showed that mortality significantly increased when GFR <61.7 ml/(min•1.73 m^2^). There were 2 missing data for GFR in the survival group.

Kaplan-meier curve analysis was performed on NT-proBNP and GFR, respectively according to the optimal prediction points of NT-proBNP and GFR obtained by ROC curve as the tangent value (Figure 2), which showed that mortality significantly increased when NT-proBNP > 2,137 pg/ml and GFR < 61.7 ml/(min•1.73 m^2^). Two groups of variables were divided into the following four groups:

A: NT-proBNP ≤ 2,137 pg/ml, GFR ≥ 61.7 ml/(min•1.73 m^2^);
B: NT-proBNP ≤ 2,137 pg/ml, GFR <61.7 ml/(min•1.73 m^2^);
C: NT-proBNP > 2,137 pg/ml, GFR ≥ 61.7 ml/(min•1.73 m^2^);
D: NT-proBNP > 2,137 pg/ml, GFR <61.7 ml/(min•1.73 m^2^).

**Figure 3:**
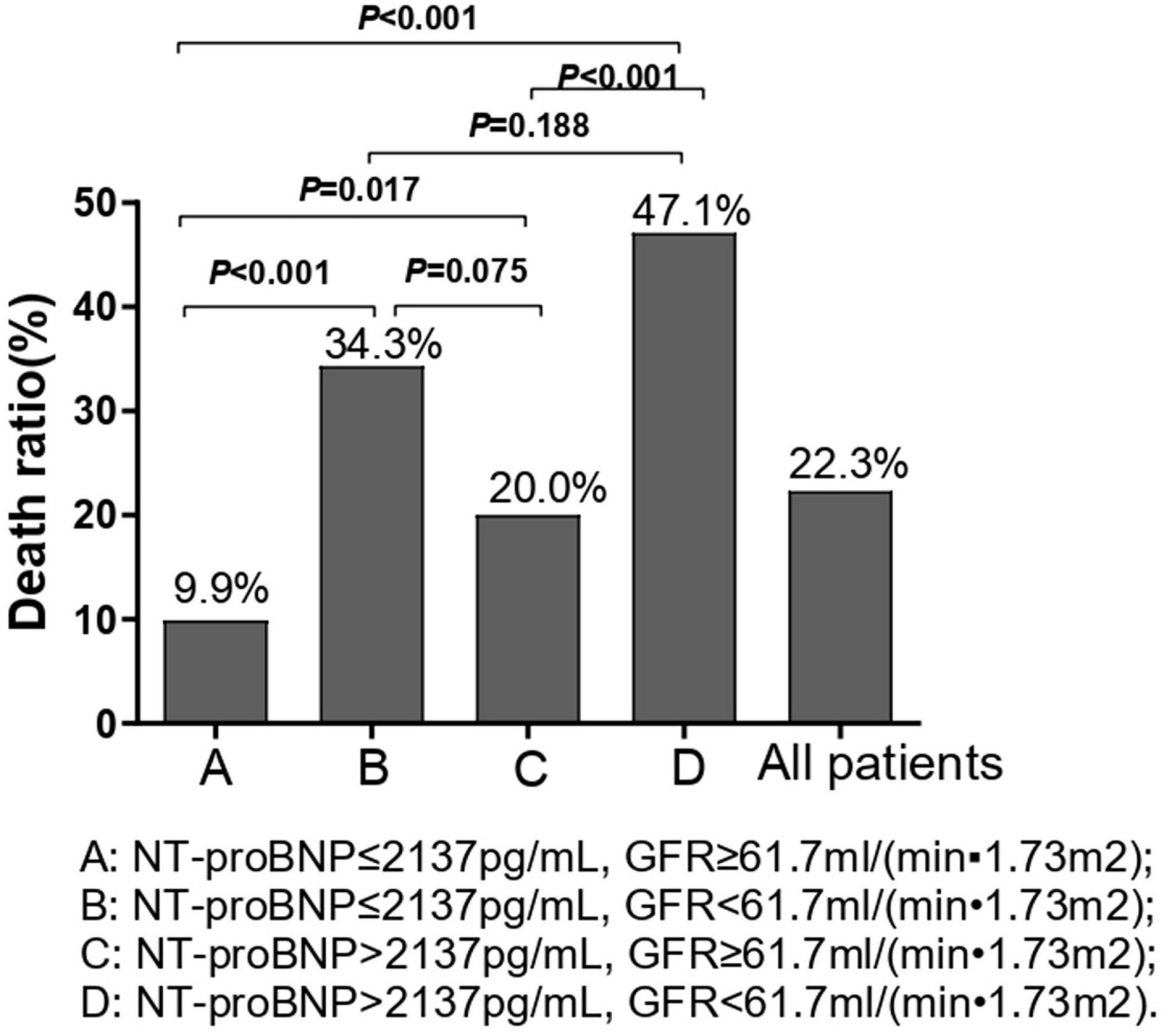
Chi-square test was performed on the four groups of variables and pairwise comparisons were calculated. We used the optimal prediction points of NT-proBNP and GFR obtained by ROC curve as the criteria to divide groups in the figure. A: NT-proBNP ≤ 2,137 pg/ml, GFR ≥ 61.7 ml/(min•1.73 m^2^); B: NT-proBNP ≤ 2,137 pg/ml, GFR <61.7 ml/(min•1.73 m^2^); C: NT-proBNP > 2,137 pg/ml, GFR ≥ 61.7 ml/(min•1.73 m^2^); D: NT-proBNP > 2,137pg/ml, GFR <61.7 ml/(min•1.73 m^2^).

**Figure 4:**
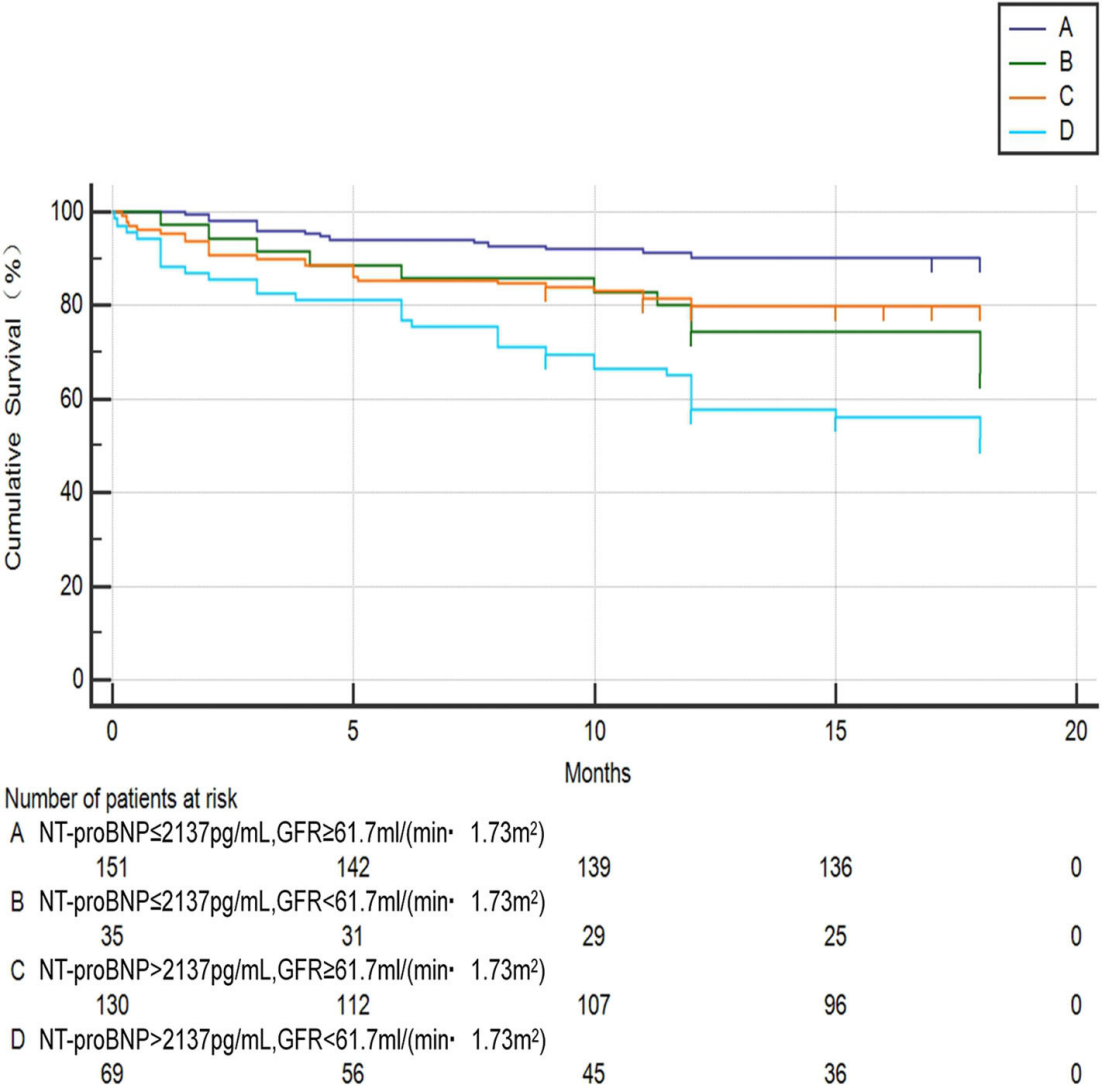
Kaplan-meier curve analysis for predicting survival status of AHF patients between the four groups. A: NT-proBNP ≤ 2,137 pg/ml, GFR ≥ 61.7 ml/(min•1.73 m^2^); B: NT-proBNP ≤ 2,137 pg/ml, GFR <61.7 ml/(min•1.73 m^2^); C: NT-proBNP > 2,137 pg/ml, GFR ≥ 61.7 ml/(min•1.73 m^2^); D: NT-proBNP > 2,137 pg/ml, GFR <61.7 ml/(min•1.73 m^2^).

Chi-square test was performed on the four groups of variables and pairwise comparisons were calculated (Figure 3). The mortality rates of groups A, B, C, and D were 9.9, 34.3, 20.0, and 47.1%, respectively. Group A was statistically lower compared with group B, C, and D, and the difference between group C and group D was statistically significant, while that between group B and group D was insignificant (*P* = 0.188), which showed that mortality had no significant difference between NT-proBNP > 2,137 pg/ml and NT-proBNP <2,137 pg/ml when GFR <61.7 ml/ (min•1.73 m^2^). In order to further compare and analyze the Kaplan-Meier survival curve among the four groups which was used to predict survival of patients with AHF at 18 months (Figure 4 and Table 3), it was found that the risk of death was more than 2 times higher in group B than in group A (HR = 3.71, 95% CI: 1.71–8.03), group D was more than 1 times higher compared with group C (HR = 2.69, 95% CI: 1.41–5.15). Namely, with similar NT-proBNP level, patients with lower GFR had significantly higher risk of death. The risk of death in group D was up to 4 times higher than that in group A (HR = 5.93, 95%CI: 3.17–11.10).

**Table 3:** Hazard ratio of different groups according to NT-proBNP and GFR levels (Kaplan-Meier method).

**HR**	**A**	**B**	**C**	**D**
**(95%CI)**				
A	–	3.71^*^	2.20^*^	5.93^*^
		(1.7–8.03)	(1.35–3.60)	(3.17–11.10)
B	0.27^*^	–	0.59	1.60
	(0.12–0.58)		(0.27–1.31)	(0.66–3.86)
C	0.45^*^	1.68	–	2.69^*^
	(0.28–0.74)	(0.76–3.71)		(1.41–5.15)
D	0.17^*^	0.62	0.37^*^	–
	(0.09–0.32)	(0.26–1.51)	(0.19–0.71)	

*The data in the table were HR values and 95% confidence intervals compared between each group*.

* *represented comparison between groups P <0.05*.

## Discussion

NT-proBNP is a focus in the study of HF, which effects on blood vessels, heart, and kidney. NT-proBNP has the effects on increasing GFR, inducing diuresis, reversing ventricular remodeling and reducing angiotasis. Based on its pathophysiology characteristic, level of brain natriuretic peptide can be used to guide the clinical medication, such as lyophilized recombinant human brain natriuretic peptide (rh-BNP) (23). Due to its ability to lower the excitability of sympathetic nervous system, this peptide can relax vascular smooth muscle, decrease blood pressure, and reduce cardiac afterload. In addition, its vasoconstriction against the renin-angiotensin-aldosterone system (RAAS) can inhibit the secretion of renin, dilate the arteries, increase the renal blood flow, and produce strong diuretic and natriuretic effects, so as to relieve the symptoms of AHF. In conclusion, NT-proBNP has become a recognized biomarker for diagnosis and prognosis of HF (24), and had a certain predictive value for the risk assessment of death in patients with atrial fibrillation (25). In addition, NT-proBNP also has a high value of negative prediction. HF can be almost excluded when NT-proBNP is normal.

Heart and kidney interact with each other through various mechanisms, including hemodynamic abnormalities, activation of neurohormones and inflammatory activities, oxidative stress, anemia, renal sympathetic nerve activity, and vitamin D metabolism, etc. In the progression of cardiorenal syndrome, impaired cardiac function may be based on the following aspects:(1) In the case of renal dysfunction, the kidney usually fails to excrete sodium properly, which results in water-sodium retention, elevated blood pressure, increased cardiac preload, and ultimately aggravates pulmonary congestion and HF. (2) HF is easy to induce hypohemoglobinemia. In severe renal dysfunction, insufficient erythropoietin will aggravate anemia, speed up heart rate compensation, strengthen myocardial contractility, activate sympathetic nerves, constrict pulmonary vessels, increase pulmonary artery pressure, and consequently aggravate cardiac remodeling.

AHF, renal insufficiency and anemia interact in a vicious circle known as cardiac anemia syndrome. The mechanism of AHF injury to renal function is considered as follows: (1) with the development of acute heart pump failure and severe water-sodium retention, the kidneys will have insufficient circulating blood volume and impaired self-regulation function. Meanwhile, hypoxia and endotoxin will cause renal vasoconstriction and further reduce renal perfusion. Then, continuous hypoperfusion can increase the susceptibility of kidney to various risk factors, which results in renal unit necrosis and apoptosis, and leads to renal cortical ischemia and infarction (19). (2) The release of inflammatory cytokines such as tumor necrosis factor-alpha, interleukin-1, and interleukin-6 leads to chronic injury and apoptosis of kidney cells, and ultimately aggravates deterioration of renal function (26). In addition, both AHF and renal insufficiency are prone to electrolyte disturbances during progression, which can disrupt homeostasis balance in the body and further worsen the disease.

The results of this study are consistent with the previous reported predictive value of NT-proBNP for the prognosis of AHF. It showed that univariate analysis of NT-proBNP had profound statistical significance. Its high sensitivity and low specificity could be due to the fact that plasma NT-proBNP is mainly metabolized in the kidney. When the kidney is damaged, the decrease of GFR further promotes the increase of serum NT-proBNP. Moreover, since NT-proBNP is affected by many factors such as race, gender, anemia, obesity, and atrial fibrillation, simple detection of NT-proBNP for diagnosis could lead to misdiagnosis of etiology (27), which also explains why NT-proBNP can be interfered by other factors in multi-factor analysis. Therefore, renal function and other related factors must be considered when using NT-proBNP as cardiac biomarker. Related studies on nephrology have found that NT-proBNP is also of certain value in the diagnosis of renal function level and can assist in predicting the prognosis of patients with middle and advanced renal insufficiency (28).

This study also suggested that GFR can be used as a powerful biomarker for predicting long-term cardiovascular events in patients with AHF after discharge. GFR has a high specificity in predicting the prognosis of AHF (81.0%). Its optimal predictive value is 61.7 ml/(min•1.73 m^2^), which is close to the classification of moderate renal insufficiency. GFR, the best comprehensive index to evaluate renal function, was significantly increased its predictive value and accuracy after combination with NT-proBNP, and additional information can be added in combined analysis (sensitivity 53.5%, specificity 85.3%). A study have suggested that patients with severe renal insufficiency (GFR <30 ml/min/1.73 m^2^) account for 30% of acute systolic HF cases and GFR was independent predictor of 1-year mortality in the community (29). In addition to the certain prediction effect of prognosis of HF, large sample statistics have found that the reduction of GFR could also lead to increased risk of atrial fibrillation events, and its evaluation value was higher when combined with NT-proBNP (30). In conclusion, timely detection and diagnosis of renal insufficiency during hospitalization are of great significance to the prognosis of patients. Although prerenal renal injury is mainly caused by renal insufficiency and it is possible to recover after timely replenishment of circulating blood volume, this study showed that the mortality rate of such patients is still high. During prerenal injury, the factors of cardiogenic renal insufficiency cannot be modified quickly, and the improvement of cardiac function requires a long time, therefore the effective circulation capacity of the kidney cannot be guaranteed, which affects the recovery of renal function and the prognosis of patients.

Because of the interaction between NT-proBNP and GFR, both of them should be considered when assessing the long-term survival risk assessment of patients. In this study, the optimal prediction value of NT-proBNP and GFR was the cut-off point for grouping comparison. In the grouping comparison, there were statistically significant differences in GFR between the two groups based on the classification of NT-proBNP. In particular, the 18-month all-cause mortality was significantly increased in patients in group D (NT-proBNP > 2,137 pg/ml, GFR <61.7 ml/(min•1.73 m^2^). In the GFR-based classification, for patients with GFR > 61.7 ml/(min•1.73 m^2^), there were statistically significant differences in the mortality between the two groups with different NT-proBNP. However, for patients with GFR <61.7 ml/min. 1.73 m^2^), there were no significant differences in mortality between the two groups separated by NT-proBNP (cut off point is 2,137 pg/ml), which suggested that for patients with moderate or above renal insufficiency, the concentration of NT-proBNP were susceptible to the GFR, and it could be indespensible to adopt combined judgment. The all-cause mortality rates in both groups were higher than those in the two groups with relatively preserved renal function, which further confirmed the high predictive value of GFR, therefore the combined evaluation could more accurately reflect the risk of death in patients with AHF.

Overall, this study investigated the effect of NT-proBNP and GFR on long-term prognosis and risk stratification in patients with AHF. The results showed that both GFR and NT-proBNP were significantly correlated with the risk of death in AHF patients. Moreover, the combination of these two values was of higher predictive value for the long-term prognosis of patients with AHF. This discovery could increase clinicians' attention to renal function in patients with AHF, which is helpful for adjusting the therapeutic regimen and taking effective intervention in patients with renal insufficiency.

## Study Limitations

This study has the following shortcomings: (1) this study is a single-center study, which may have population bias. Currently, multi-center studies have been carried out synchronously. The objective is to collect data in 2,000 AHF patients and establish a database, the results of this study will be confirmed by larger sample size population data analysis in the later stage. (2) In this study, we also found that SBP, sodium, hemoglobin, and PASP were independent predictors of all-cause mortality at 18 months, which is consistent with previously reported findings. However, the prognosis of hospitalized patients with HF is still limited. More new predictors need to be discovered and the joint prediction of multiple factors should be widely used in future studies. (3) GFR is now considered as the best comprehensive indicator of renal function, but it is only a part of renal function, and CKD-EPI formula has some limitations. However, it is difficult to use inulin or iodide phthalate clearance rate to calculate accurate GFR in clinical work. At present, CKD-EPI_CysC_ formula based on cystatin is still applicable according to the kidney disease guidelines and its accuracy has been well-recognized in clinical trials. Due to the lack of cystatin data in this experiment, the applicability of this formula could not be verified. Instead, CKD-EPI_SCR_ formula based on serum creatinine, which is widely used in clinical practice and has high international recognition. CKD-EPI is closer to the accurate value compared with the previous MDRD algorithm (31).

## Conclusion

In patients with NT-proBNP > 2,137 pg/ml and GFR <61.7 ml/(min•1.73 m^2^), the risk of death is significantly higher. The combination of GFR and NT-proBNP improves the predictive value for the long-term prognosis of AHF patients. Early identification of these high-risk patients could help clinicians to modify and strengthen treatment regimens, thereby improving the clinical prognosis of these patients.

## Data Availability Statement

The raw data supporting the conclusions of this article will be made available by the authors, without undue reservation.

## Ethics Statement

The studies involving human participants were reviewed and approved by Ethics committee of the first affiliated Hospital of Nanjing Medical University. The patients/participants provided their written informed consent to participate in this study.

## Author Contributions

XL and HZ: study design and interpretation of results. KW, QW, GN, YZ, and WY: data collection. KW, QW, and GN: data analysis. KW, XL, and HZ: preparation of manuscript. KW, GN, QW, XL, and HZ: revision of manuscript. All authors contributed to the article and approved the submitted version.

### Conflict of Interest

The authors declare that the research was conducted in the absence of any commercial or financial relationships that could be construed as a potential conflict of interest.
